# Risk factors associated with suicide among kidney cancer patients: A Surveillance, Epidemiology, and End Results analysis

**DOI:** 10.1002/cam4.2400

**Published:** 2019-07-11

**Authors:** Chenyu Guo, Wenwen Zheng, Weiwei Zhu, Shengqiang Yu, Yuexia Ding, Qingna Wu, Qiling Tang, Congxiao Lu

**Affiliations:** ^1^ Department of Pharmacy Yantai Yuhuangding Hospital Yantai China; ^2^ Department of Urology Yantai Yuhuangding Hospital Yantai China

**Keywords:** kidney cancer, risk factors, SEER, suicide

## Abstract

**Background:**

The suicide risk was higher in kidney cancer patients than in the general population. The purpose of this study was to characterize the suicide rates among kidney cancer patients and to identify the potential risk factors associated with suicide from the Surveillance, Epidemiology, and End Results (SEER) database.

**Methods:**

Kidney cancer patients were identified from the SEER database during 1973‐2015. Suicide rates and standardized mortality ratios (SMRs) of this population were calculated, and the US general population during 1981‐2015 was chosen as a reference. Univariable and multivariable Cox regression were performed to find out potential risk factors of suicide.

**Results:**

There were 207 suicides identified among 171 819 individuals with kidney cancer observed for 948 272 person‐years. The suicide rate was 21.83 per 100 000 person‐years, and SMR was 1.83 (95% CI: 1.59‐2.10). On Cox regression, diagnosis in early years (1973‐1982 vs 2003‐2015, HR: 2.03, 95% CI: 1.01‐4.11, *P* = 0.048; 1983‐1992 vs 2003‐2015, HR: 1.99, 95% CI: 1.18‐3.35, *P* = 0.010), male sex (vs female sex, HR: 4.43, 95% CI: 2.95‐6.65, *P* < 0.001), unmarried status (vs married status, HR: 2.54, 95% CI: 1.91‐3.38, *P* < 0.001), non‐black race (white race vs black race, HR: 4.47, 95% CI: 2.09‐9.58, *P* < 0.001; other races vs black race, HR: 3.01, 95% CI: 1.08‐8.37, *P* = 0.035), higher histologic grade (grade IV vs grade I, HR: 3.27, 95% CI: 1.50‐7.13, *P* = 0.003; grade III vs grade I, HR: 2.13, 95% CI: 1.19‐3.81, *P* = 0.011) and cancer‐directed surgery not performed (vs performed, HR: 2.78, 95% CI: 1.52‐5.11, *P* < 0.001) were independent risk factors of suicide among kidney cancer patients.

**Conclusions:**

Diagnosis in early years, male sex, unmarried status, non‐black race, higher histologic grade, and cancer‐directed surgery not performed were significantly associated with suicide among kidney cancer patients. In order to prevent suicidal death, clinicians should pay more attention to patients with high‐risk factors of suicide.

## 1 BACKGROUND

Suicide, a global public health problem, was a complex behavior affecting by physiological, psychological, social, environmental, and cultural factors.^1^ World Health Organization reported that over 800 000 persons committed suicide every year and one person every 40 seconds.^1^ Globally, 81 7000 persons died of suicide in 2016, comprising 1.49% of total deaths.^2^ In the US, the suicide mortality rate was 15.3 per 100 000 in 2016, which was relatively higher in comparison with the surrounding countries.^3^

In recent years, studies reported a notable association between depression and suicide, and the ssuicide rate among patients with depression was much higher than the usual population.^4^, ^5^, ^6^ The patients diagnosed as diseases with poor prognosis, especially cancer, were more likely to feel hopeless, suffer from depression and subsequently commit suicide.^7^, ^8^ Several studies reported increased suicide ideation and attempts in patients with cancer and showed a high suicide rate among these patients.^7^, ^9^, ^10^ Compared with the general population, the suicide rate of patients with cancer was almost twice in the US.^11^ A current study by Zaorsky et al revealed that the cancer patients had a suicide SMR of 4.44 in comparison with the general population.^12^ For the behavior of suicide could be potentially recognized and prevented, the identification of patients with high‐risk factors of suicide was particularly important. The risk factors including male sex, white race, and unmarried status were demonstrated to be associated with a high incidence of suicide for some types of cancer, such as breast cancer, colorectal cancer, and pancreatic cancer.^13^, ^14^, ^15^

Kidney cancer was the seventh and the ninth most common malignancy in men and women.^16^ In the US, the number of patients with kidney cancer was estimated to be 65 340 in 2018, and the number of death cases was estimated to be 14 970.^17^ Misono et al reported increased suicide rates of patients with cancer in the US and characterized this specific population.^11^ However, the risk factors of suicide in patients with kidney cancer were not involved in the study. Klaassen et al selected the patients with kidney cancer through the SEER database and identified the risk factors of suicide.^18^ However, papillary renal cell carcinoma was not included in this study, which was the second most frequency subtype in all renal cortical neoplasms.^19^ Histologic subtype was an important prognostic factor for kidney cancer survival.^20^ To our best knowledge, there was no previous study to investigate the association between histologic subtype and suicide among patients with kidney cancer. Additionally, few studies explored the risk factors associated with suicide among kidney cancer patients based on a large representative sample. Therefore, the purpose of our study was to characterize the suicide rates and SMRs in this specific cohort and to identify the potential risk factors relevant to suicide based on the SEER database.

## 2 METHODS

### 2.1 Data source

All patients with kidney cancer were selected from the SEER database (1973‐2015). The SEER database reported cancer‐specific outcomes from specific geographic areas covering 28% of the US population. The data in the SEER program were considered to be representative of the entire US population.^21^ The SEER database could freely provide information including patient demographics, cancer incidence, and survival data to registered researchers. The permission of accessing the database was obtained after we signed and submitted a SEER Research Data Agreement form through e‐mail. The software of SEER*Stat (version 8.3.5) was used to identify the patients.

### 2.2 Study population

Patients were identified using the primary site codes (C64.9 and C65.9) for kidney and morphology codes (8050/3, 8260/3, 8310/3, 8312/3, 8313/3, 8316/3, 8317/3, 8318/3, and 8319/3) based on International Classification of Diseases for Oncology codes (3rd edition) for kidney cancer.

### 2.3 Measurements of variables

**Figure 1:**
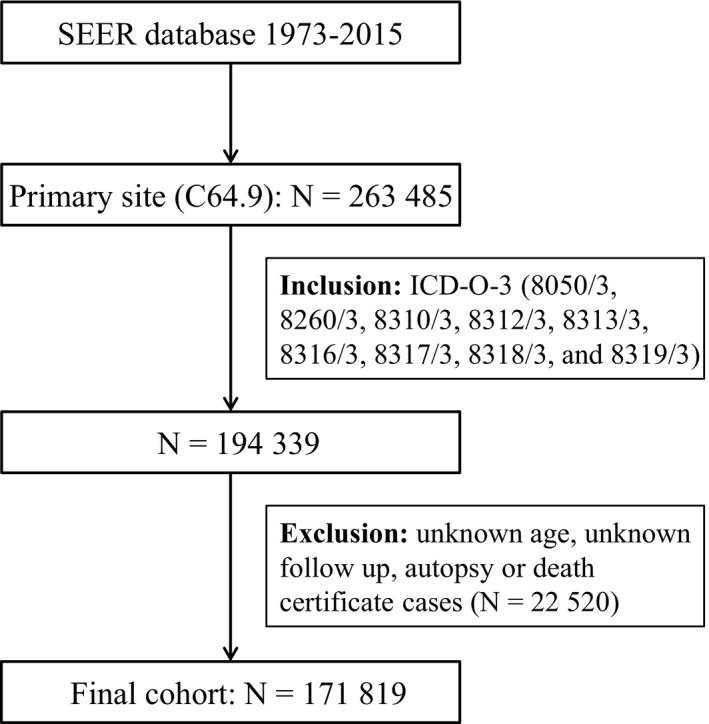
The flow diagram of patient selection

Demographic and clinical variables of interest were collected through the software of SEER*Stat, including year of diagnosis (1973‐1982, 1983‐1992, 1993‐2002, 2003‐2015), age at diagnosis (≤39, 40‐49, 50‐59, 60‐69, 70‐79, ≥80), gender (male, female), marital status (married, unmarried), race (black, white, others), SEER histologic stage (localized, regional, distant), histologic grade (well differentiated; grade I, moderately differentiated; grade II, poorly differentiated; grade III, undifferentiated; anaplastic; grade IV), histologic subtype (clear renal cell carcinoma, papillary renal cell carcinoma, chromophobe renal cell carcinoma, sarcomatoid renal cell carcinoma, collecting duct renal cell carcinoma, others), survival time, vital status, surgery (yes, no), and radiotherapy (yes, no). Based on marital status, the patients were classified as married and unmarried, and the unmarried status included single, widowed, divorced/separated, and unmarried or domestic partner. The exclusion criteria included patients with unknown age, unknown follow‐up, and all autopsy or death certificate cases. The steps of patient selection were exhibited in Figure 1.

### 2.4 Ascertainment of the outcome

The primary outcome of this study was suicidal death, which was ascertained by cause of death code (suicide and self‐inflicted injury) in the software of SEER*Stat.

### 2.5 Statistical analysis

The suicide rates between groups were compared with the chi‐square test, and the Bonferroni‐corrected *P*‐value was used for multiple comparisons. SMR was calculated using the ratio of actual mortality with expected mortality. Because of the lack of suicide rates during 1973‐1980 in the Centers for Disease Control and Prevention's Web‐based Injury Statistics Query and Reporting System, the US general population between 1981 and 2015 was chosen as a reference, and a sensitive analysis with the kidney cancer patients diagnosed in 1981‐2015 was performed to validate the robustness of SMRs. Ninety‐five percent of CI of SMR was obtained through the method of Byar's approximation.^22^ Univariable and multivariable Cox regression were performed to generate crude and adjusted HRs and 95% CI for finding out potential risk factors of suicide. The proportional hazards assumption of Cox regression modeling was evaluated with the use of Schoenfeld residuals. All statistical analysis was carried out using R software (version 3.5.2, http://www.r-project.org/). All tests were two‐sided, and the significance level was set at *P* < 0.05.

## 3 RESULTS

### 3.1 Patient baseline characteristics

Overall, 171 819 patients with kidney cancer were identified from the SEER database in 1973‐2015, including 109 810 male patients and 62 009 female patients. Of these, 207 patients (0.1%) died of suicide, 69 182 patients (40.3%) died of other causes, and 102 430 patients (59.6%) were alive. Among all patients, 107 623 (62.6%) of them were married, while 56 515 (32.9%) of them were unmarried. Caucasian was the predominant race. A total of 154 139 (89.7%) patients underwent cancer‐directed surgery and 167 176 (97.3%) patients did not receive radiotherapy. For the patients committed suicide, 179 (86.5%) of them were males, and 28 (13.5%) of them were females. Concerning marital status, 110 (53.1%) of them were married, whereas 92 (44.4%) of them were unmarried. Similarly, Caucasian was also the predominant race. 189 (91.3%) patients received cancer‐directed surgery, and only 4 (1.9%) patients received radiotherapy. Patient demographics and clinical characteristics were summarized in Table 1.

**Table 1:** Baseline characteristics of patients with kidney cancer stratified by suicidal death, nonsuicidal death and alive (1973‐2015)

Variables	Overall N (%)	Suicidal death N (%)	Nonsuicidal death N (%)	Alive N (%)
Patients	171 819	207	69 182	102 430
Year of diagnosis				
1973‐1982	4394 (2.6%)	11 (5.3%)	4149 (6.0%)	234 (0.2%)
1983‐1992	8968 (5.2%)	27 (13.0%)	7740 (11.2%)	1201 (1.2%)
1993‐2002	30 556 (17.8%)	45 (21.7%)	20 113 (29.1%)	10 398 (10.2%)
2003‐2015	127 901 (74.4%)	124 (59.9%)	37 180 (53.7%)	90 597 (88.4%)
Sex				
Male	109 810 (63.9%)	179 (86.5%)	45 886 (66.3%)	63 745 (62.2%)
Female	62 009 (36.1%)	28 (13.5%)	23 296 (33.7%)	38 685 (37.8%)
Age at diagnosis				
≤39	8027 (4.7%)	6 (2.9%)	1359 (2.0%)	6662 (6.5%)
40‐49	21 281 (12.4%)	32 (15.5%)	5378 (7.8%)	15 871 (15.5%)
50‐59	41 790 (24.3%)	51 (24.6%)	13 692 (19.8%)	28 047 (27.4%)
60‐69	50 656 (29.5%)	59 (28.5%)	20 332 (29.4%)	30 265 (29.5%)
70‐79	37 206 (21.7%)	45 (21.7%)	19 798 (28.6%)	17 363 (17.0%)
≥80	12 859 (7.5%)	14 (6.8%)	8623 (12.5%)	4222 (4.1%)
Marital status				
Married	107 623 (62.6%)	110 (53.1%)	41 999 (60.7%)	65 514 (64.0%)
Unmarried	56 515 (32.9%)	92 (44.4%)	24 726 (35.7%)	31 697 (30.9%)
Unknown	7681 (4.5%)	5 (2.4%)	2457 (3.6%)	5219 (5.1%)
Race				
White	140 915 (82.0%)	192 (92.8%)	57 204 (82.7%)	83 519 (81.5%)
Black	20 107 (11.7%)	7 (3.4%)	8294 (12.0%)	11 806 (11.5%)
Other	9822 (5.7%)	8 (3.9%)	3615 (5.2%)	6199 (6.1%)
Unknown	975 (0.6%)	0 (0.0%)	69 (0.1%)	906 (0.9%)
Histologic grade				
Grade I	18.372 (10.7%)	16 (7.7%)	5758 (8.3%)	12 598 (12.3%)
Grade II	62 462 (36.4%)	67 (32.4%)	16 631 (24.0%)	45 764 (44.7%)
Grade III	33 431 (19.5%)	45 (21.7%)	12 499 (18.1%)	20 887 (20.4%)
Grade IV	8182 (4.8%)	12 (5.8%)	4839 (7.0%)	3331 (3.3%)
Unknown	49 372 (28.7%)	67 (32.4%)	29 455 (42.6%)	19 850 (19.4%)
SEER disease stage				
Localized	117 927 (68.6%)	151 (72.9%)	33 243 (48.1%)	84 533 (82.5%)
Regional	28 531 (16.6%)	33 (15.9%)	14 661 (21.2%)	13 837 (13.5%)
Distant	22 697 (13.2%)	19 (9.2%)	19 560 (28.3%)	3118 (3.0%)
Unstaged	2664 (1.6%)	4 (1.9%)	1718 (2.5%)	942 (0.9%)
Histologic subtype^a^				
cRCC	86 790 (50.5%)	83 (40.1%)	27 253 (39.4%)	59 454 (58.0%)
pRCC	18 119 (10.5%)	24 (11.6%)	4886 (7.1%)	13 209 (12.9%)
chRCC	7388 (4.3%)	10 (4.8%)	1132 (1.6%)	6246 (6.1%)
sRCC	2172 (1.3%)	2 (1.0%)	1734 (2.5%)	436 (0.4%)
cdRCC	412 (0.2%)	0 (0.0%)	292 (0.4%)	120 (0.1%)
Others	56 938 (33.1%)	88 (42.5%)	33 885 (49.0%)	22 965 (22.4%)
Surgery performed				
Yes	154 139 (89.7%)	189 (91.3%)	54 842 (79.3%)	99 108 (96.8%)
No	16 904 (9.8%)	17 (8.2%)	13 766 (19.9%)	3121 (3.0%)
Unknown	776 (0.5%)	1 (0.5%)	574 (0.8%)	201 (0.2%)
Radiotherapy performed				
Yes	4643 (2.7%)	4 (1.9%)	3916 (5.7%)	723 (0.7%)
No	167 176 (97.3%)	203 (98.1%)	65 266 (94.3%)	101 707 (99.3%)

a cRCC, clear renal cell carcinoma; pRCC, papillary renal cell carcinoma; chRCC, chromophobe renal cell carcinoma; sRCC, sarcomatoid renal cell carcinoma; cdRCC, collecting duct renal cell carcinoma.

### 3.2 Difference in suicide rates and SMRs

From 1973 to 2015, there were 207 suicides among 171 819 patients with kidney cancer observed for 948 272 person‐years, yielding a suicide rate of 21.83 per 100 000 person‐years. The suicide rate of US general population reported by the Centers for Disease Control and Prevention was 11.93 per 100 000 person‐years between 1981 and 2015,^23^ which was significantly lower than that of the kidney cancer patients in our cohort (*P* < 0.001). The results showed that higher suicide rates in patients with kidney cancer were associated with male sex (vs female, *P* < 0.001), unmarried status (vs married, Bonferroni‐corrected *P* < 0.001), white race (vs black race, Bonferroni‐corrected *P* = 0.002), and cancer‐directed surgery not performed (vs performed, Bonferroni‐corrected *P* < 0.001). The result of the chi‐square test for linear trend showed that suicide rates among kidney cancer patients increased with histologic grade (*P* < 0.001) and disease stage (*P* < 0.001). Meanwhile, no statistical differences in suicide rates were found with respect to year of diagnosis, age at diagnosis, histologic type, radiotherapy, and time from diagnosis. SMRs were used to compare suicide mortality in the study population to that in the general population. A SMR of 1.83 (95% CI: 1.59‐2.10) was noted between kidney cancer patients and the US general population, with 1.57 (95% CI: 1.35‐1.82) for males, 1.60 (95% CI: 1.06‐2.31) for females, 1.85 (95% CI: 1.60‐2.13) for the white race, 1.14 (95% CI: 0.46‐2.35) for the black race, and 2.19 (95% CI: 0.94‐4.31) for other races. A general decline of suicide rates over time after diagnosis was observed, though a statistical trend was not found (*P* = 0.064). Significantly increased suicide rates among kidney cancer patients with general population were found in the initial 9 years after cancer diagnosis (0‐3 years SMR: 2.04, 95% CI: 1.64‐2.49; 4‐6 years SMR: 1.76, 95% CI: 1.31‐2.32; 7‐9 years SMR: 1.98, 95% CI: 1.38‐2.75). The suicide rates and SMRs of kidney cancer patients diagnosed in 1973‐2015 and 1981‐2015 were presented in Table 2 and Supplementary Table 1, respectively. The sensitivity analysis exhibited good robustness of SMRs.

**Table 2:** Suicide rates and SMRs among patients with kidney cancer by demographic and clinic characteristics (1973‐2015)

Variables	Suicidal death	Person‐years	Suicide rate per 100 000 person‐years	*P*	SMR^a^	95% CI
Total	207	948 272	21.83	‐	1.83	1.59‐2.10
Year of diagnosis						
1973‐1982	11	41 401	26.57	0.08	2.23	1.11‐3.99
1983‐1992	27	89 349	30.22		2.53	1.67‐3.69
1993‐2002	45	269 896	16.67		1.40	1.02‐1.87
2003‐2015	124	547 626	22.64		1.90	1.58‐2.26
Sex						
Male	179	591 101	30.28	**<0.001** ***	1.57	1.35‐1.82
Female	28	357 171	7.84		1.60	1.06‐2.31
Age at diagnosis						
≤39	6	59 155	10.14	0.224	0.85	0.31‐1.85
40‐49	32	146 966	21.77		1.83	1.25‐2.58
50‐59	51	252 419	20.20		1.69	1.26‐2.23
60‐69	59	268 947	21.94		1.84	1.40‐2.37
70‐79	45	175 964	25.57		2.14	1.56‐2.87
≥80	14	44 821	31.24		2.62	1.43‐4.39
Marital status						
Married	110	627 490	17.53	**<0.001** ***	1.47	1.21‐1.77
Unmarried	92	282 904	32.52		2.73	2.20‐3.34
Unknown	5	37 878	13.20		1.11	0.36‐2.58
Race						
White	192	783 889	24.49	**<0.001** ***	1.85	1.60‐2.13
Black	7	107 206	6.53		1.14	0.46‐2.35
Others	8	52 750	15.17		2.19	0.94‐4.31
Unknown	0	4427	0		‐	‐
Histologic grade						
Grade I	16	126 328	12.67	**0.001** **	1.06	0.61‐1.72
Grade II	67	355 237	18.86		1.58	1.23‐2.01
Grade III	45	149 563	30.09		2.52	1.84‐3.37
Grade IV	12	24 889	48.21		4.04	2.09‐7.06
Unknown	67	292 255	22.93		1.92	1.49‐2.44
SEER disease stage						
Localized	151	753 396	20.04	**<0.001** ***	1.68	1.42‐1.97
Regional	33	148 040	22.29		1.87	1.29‐2.62
Distant	19	34 591	54.93		4.60	2.77‐7.19
Unstaged	4	12 245	32.67		2.74	0.74‐7.01
Histologic subtype^b^						
cRCC	83	431 537	19.23	0.299	1.61	1.28‐2.00
pRCC	24	87 208	27.52		2.31	1.48‐3.43
chRCC	10	37 833	26.43		2.22	1.06‐4.07
sRCC	2	3844	52.03		4.36	0.49‐15.75
cdRCC	0	1323	0		‐	‐
Others	88	386 527	22.77		1.91	1.53‐2.35
Surgery performed						
Yes	189	922 663	20.48	**<0.001** ***	1.72	1.48‐1.98
No	17	23 950	70.98		5.95	3.46‐9.53
Unknown	1	1659	60.28		5.05	0.07‐28.11
Radiotherapy performed						
Yes	4	12 921	30.96	0.684	2.59	0.70‐6.64
No	203	935 351	21.70		1.82	1.58‐2.09
Time from diagnosis						
0‐3 years	93	382 855	24.29	0.308	2.04	1.64‐2.49
4‐6 years	51	242 432	21.04		1.76	1.31‐2.32
7‐9 years	35	148 130	23.63		1.98	1.38‐2.75
10‐12 years	16	83 341	19.20		1.61	0.92‐2.61
>13 years	12	91 514	13.11		1.10	0.57‐1.92

The P and HR values in the bold are statistically significant.

a Compared with the suicide rates of the general US population based on the Centers for Disease Control and Prevention's Web‐based Injury Statistics Query and Reporting System (1981‐2015).

b cRCC, clear renal cell carcinoma; pRCC, papillary renal cell carcinoma; chRCC, chromophobe renal cell carcinoma; sRCC, sarcomatoid renal cell carcinoma; cdRCC, collecting duct renal cell carcinoma.

** *P* < 0.01,

*** *P* < 0.001.

### 3.3 Associations of risk factors with suicide

The result of univariable analysis presented that significant associations with high risks of suicide were obtained with respect to diagnosis in 1983‐1992 (vs 2003‐2015, HR: 1.61, 95% CI: 1.03‐2.51, *P* < 0.001), male sex (vs female sex, HR: 3.84, 95% CI: 2.58‐5.72, *P* < 0.001), older age at diagnosis (70‐79 vs ≤ 39, HR: 2.39, 95% CI: 1.02‐5.61, *P* = 0.046; ≥80 vs ≤ 39, HR: 2.83, 95% CI: 1.08‐7.40, *P* = 0.034), unmarried status (vs married status, HR: 1.83, 95% CI: 1.39‐2.41, *P* < 0.001), white race (vs black race, HR: 3.76, 95% CI: 1.77‐8.00, *P* < 0.001), higher histologic grade (grade IV vs grade I, HR: 3.60, 95% CI: 1.70‐7.63, *P* < 0.001; grade III vs grade I, HR: 2.29, 95% CI: 1.29‐4.05, *P* = 0.005), later disease stage (distant vs localized, HR: 2.53, 95% CI: 1.55‐4.13, *P* < 0.001), and cancer‐directed surgery not performed (vs performed, HR: 3.19, 95% CI: 1.92‐5.30, *P* < 0.001). On multivariable Cox regression, the results showed that diagnosis in early years (1973‐1982 vs 2003‐2015, HR: 2.03, 95% CI: 1.01‐4.11, *P* = 0.048; 1983‐1992 vs 2003‐2015, HR: 1.99, 95% CI: 1.18‐3.35, *P* = 0.010) and male sex (vs female sex, HR: 4.43, 95% CI: 2.95‐6.65, *P* < 0.001) were predictive of suicide. Meanwhile, unmarried status (vs married status, HR: 2.54, 95% CI: 1.91‐3.38, *P* < 0.001) was a significant risk factor of suicide. In addition, the factors associated with high risks of suicide included non‐black race (white race vs black race, HR: 4.47, 95% CI: 2.09‐9.58, *P* < 0.001; other races vs black race, HR: 3.01, 95% CI: 1.08‐8.37, *P* = 0.035), higher histologic grade (grade IV vs grade I, HR: 3.27, 95% CI: 1.50‐7.13, *P* = 0.003; grade III vs grade I, HR: 2.13, 95% CI: 1.19‐3.81, *P* = 0.011), and cancer‐directed surgery not performed (vs performed, HR: 2.78, 95% CI: 1.52‐5.11, *P* < 0.001). Conversely, apparent associations of suicide with age at diagnosis, SEER disease stage, histologic subtype, and radiotherapy were not found. Table 3 presented further details about the predictors of suicide in the entire cohort.

**Table 3:** Univariable and multivariable analysis for suicide of kidney cancer patients

Variables	Univariable analysis		Multivariable analysis	
	HR(95% CI)	*P*	HR(95% CI)	*P*
Year of diagnosis				
2003‐2015	Reference		Reference	
1993‐2002	0.84 (0.59‐1.19)	0.325	0.92 (0.63‐1.34)	0.654
1983‐1992	**1.61 (1.03‐2.51)**	**0.036** *	**1.99 (1.18‐3.35)**	**0.010** *
1973‐1982	1.50 (0.79‐2.85)	0.211	**2.03 (1.01‐4.11)**	**0.048** *
Sex				
Female	Reference		Reference	
Male	**3.84 (2.58‐5.72)**	**<0.001** ***	**4.43 (2.95‐6.65)**	**<0.001** ***
Age at diagnosis				
≤39	Reference		Reference	
40‐49	2.12 (0.89‐5,07)	0.091	2.13 (0.89‐5.11)	0.089
50‐59	1.94 (0.83‐4.52)	0.126	1.96 (0.84‐4.59)	0.120
60‐69	2.08 (0.90‐4.82)	0.089	2.13 (0.91‐4.96)	0.080
70‐79	**2.39 (1.02‐5.61)**	**0.046** *	2.25 (0.98‐5.79)	0.055
≥80	**2.83 (1.08‐7.40)**	**0.034** *	2.61 (0.99‐6.87)	0.052
Marital status				
Married	Reference		Reference	
Unmarried	**1.83 (1.39‐2.41)**	**<0.001** ***	**2.54 (1.91‐3.38)**	**<0.001** ***
Unknown	0.74 (0.30‐1.82)	0.512	0.85 (0.34‐2.09)	0.720
Race				
Black	Reference		Reference	
White	**3.76 (1.77‐8.00)**	**<0.001** ***	**4.47 (2.09‐9.58)**	**<0.001** ***
Others	2.32 (0.84‐6.40)	0.104	**3.01 (1.08‐8.37)**	**0.035** *
Unknown	—	—	—	—
Histologic grade				
Grade I	Reference		Reference	
Grade II	1.46 (0.84‐2.52)	0.177	1.43 (0.82‐2.47)	0.204
Grade III	**2.29 (1.29‐4.05)**	**0.005** **	**2.13 (1.19‐3.81)**	**0.011** *
Grade IV	**3.60 (1.70‐7.63)**	**<0.001** ***	**3.27 (1.50‐7.13)**	**0.003** **
Unknown	1.85 (1.07‐3.20)	0.027	1.27 (0.72‐2.24)	0.401
SEER disease stage				
Localized	Reference		Reference	
Regional	1.11 (0.76‐1.61)	0.602	0.87 (0.59‐1.28)	0.483
Distant	**2.53 (1.55‐4.13)**	**<0.001** ***	1.44 (0.81‐2.57)	0.215
Unstaged	1.62 (0.60‐4.38)	0.339	1.05 (0.37‐3.02)	0.924
Histologic type^a^				
cRCC	Reference		Reference	
pRCC	1.41 (0.90‐2.22)	0.137	1.44 (0.91‐2.29)	0.123
chRCC	1.36 (0.70‐2.61)	0.364	1.60 (0.83‐3.11)	0.162
sRCC	2.50 (0.61‐10.20)	0.200	1.54 (0.36‐6.51)	0.557
cdRCC	—	—	—	—
Others	1.26 (0.93‐1.70)	0.144	1.18 (0.84‐1.65)	0.338
Surgery performed				
Yes	Reference		Reference	
No	**3.19 (1.92‐5.30)**	**<0.001** ***	**2.78 (1.52‐5.11)**	**<0.001** ***
Unknown	2.82 (0.40‐20.16)	0.301	2.66 (0.36‐19.77)	0.340
Radiotherapy performed				
Yes	Reference		Reference	
No	0.72 (0.27‐1.94)	0.518	1.09 (0.39‐3.06)	0.876

The P and HR values in the bold are statistically significant.

a cRCC, clear renal cell carcinoma; pRCC, papillary renal cell carcinoma; chRCC, chromophobe renal cell carcinoma; sRCC, sarcomatoid renal cell carcinoma; cdRCC, collecting duct renal cell carcinoma.

* *P* < 0.05,

** *P* < 0.01,

*** *P* < 0.001.

## 4 DISCUSSION

Several investigations from different countries reported an increased risk of suicide in the populations with cancer diagnosis.^11^, ^24^, ^25^, ^26^ Allebeck et al reported an increased suicide rate among cancer patients in Sweden, with an SMR of 1.9 for males and 1.6 for females.^24^ A study from the Danish Cancer Register revealed that the SMRs of suicide for male and female cancer patients were respectively 1.7 and 1.4 in 1971‐1999.^25^ The researchers from England and Italy also reported a similar result.^26^, ^27^ Based on our study, the results showed the suicide rate of kidney cancer patients was 21.83 per 100 000 person‐years, and SMR was 1.83 (95% CI: 1.59‐2.10), indicating an apparent increased risk of suicide in comparison with the US general population. Diagnosed in early years (1973‐1992), male sex, unmarried status, non‐black race, higher histologic grade, and cancer‐directed surgery not performed were significant predictors of suicide.

In reviewing the results, various demographic characteristics were distinctly associated with suicide risk among patients with kidney cancer. The patients diagnosed in 1973‐1992 were more likely to commit suicide in comparison with those diagnosed in 2003‐2015, and this could be possibly explained by the emergence of a new treatment and the accompanying improvement of life quality. Similarly, patients diagnosis with bladder cancer in earlier years (1973‐1983) were at significantly higher risk of suicide compared with those in recent years (2004‐2010).^28^ In the present study, the suicide rate of males was 30.28 per 100 000 person‐years, which was 2.9 times higher than that of females. Furthermore, males were more likely than females to commit suicide with an HR of 4.43, which was consistent with the findings in the general population and patients with other types of cancer.^22^, ^28^ Though it seemed that male and female cancer patients suffered similar pressure, male patients were more likely to react through self‐directed violence.^28^, ^29^, ^30^ Unmarried status was another significant risk factor of suicide with an HR of 2.54 in comparison with married status. Married patients usually had higher cancer‐specific survival and lower mortality in comparison with unmarried patients,^31^, ^32^, ^33^ which could be ascribed to better health, higher socioeconomic status, more emotional support and social attention of married persons.^34^, ^35^, ^36^ The present study corroborated the finding that race had a significant impact on suicidal death. As was reported by the Centers for Disease Control and Prevention, the black race had the lowest suicide rate among all races in the US.^37^ Our study showed that the suicide rates of the white race and other races were respectively 2.77 and 1.34 times higher than that of the black race. Furthermore, the black race was proved to be a protective factor of suicide, matching the findings in previous investigations.^13^, ^38^ In terms of the black race, this finding might be explained by religious beliefs, family support, and culture of rejecting suicide.^39^ Some recent publications showed that older age was a predictor of suicide for cancer patients and the common population.^11^, ^40^ Interestingly, older breast cancer patients who fare better in terms of functional status than younger patients were less likely to commit suicide.^13^, ^41^ In our study, a tendency of increased suicide rate with age was also observed, although without statistical association. As reported in previous studies, suicide risk among cancer patients varied by time after diagnosis, and elevated suicide rates could be found in the initial period after diagnosis.^27^, ^38^ Our findings showed significantly increased suicide rates among kidney cancer patients with the general population in the initial 9 years after diagnosis. A statistical trend of decreased suicide rates over time was not found, which might be attributed to relative better prognosis of kidney cancer in comparison with other types of cancer.

Regarding specific clinical variables of kidney cancer, several findings should be noteworthy. It was well known that low histologic grade meant well differentiated of cancer cells, predicting good prognosis and improved health‐related quality of life.^42^ The patients with higher histologic grade (Grade III and IV) in our study were found to have higher suicide risks than those with lower histologic grade (Grade I). However, this finding was contrary to the previous research by Gaitanidis that earlier disease stage and lower tumor grade were associated with higher risks of committing suicide among breast cancer patients. This conflicting result might be partially attributed to the fact that all the patients with breast cancer enrolled in the study had passed away.^13^

Increased suicide risks were commonly associated with cancer types of poor prognosis.^27^ Recently, some investigations had reported that histologic subtype was not significantly associated with suicide risks among non‐small cell lung cancer patients and colorectal cancer patients.^38^, ^43^ As we know, histologic subtype was a prognostic indicator for kidney cancer survival.^19^ Compared with clear cell renal cell carcinoma, patients with sarcomatoid renal cell carcinoma and collecting duct renal cell carcinoma had worse overall survival and cancer‐specific survival.^44^, ^45^ Conversely, patients with chromophobe histology had improved survival.^45^ Based on our study, there was no significant difference in suicide rates among patients with different histologic subtypes, and no apparent association between histologic subtype and suicide was found by Cox regression modeling. However, it should be noted that the lack of suicide event for sarcomatoid and collecting duct renal cell carcinoma which was low incidence and poor prognosis might have an effect on this result.

As shown in Table 3, another factor associated with suicide was cancer‐directed surgery. The patients underwent surgery were less likely to commit suicide than those who did not undergo surgery. Samawi et al identified the predictors of suicide in colorectal cancer patients based on the SEER database. Primary site surgery as an independent protective factor was reported in this study, probably attributing to the improvement of mortality after primary tumor resected.^14^, ^46^, ^47^ Similarly, it was reported that patients with cancers of the digestive system who underwent surgery were less likely to commit suicide.^48^ Conversely, cancer‐directed surgery was associated with increased suicide rate in breast cancer patients, which might be related to the increased adverse psychological impact after mastectomy and the relative body image disturbance.^13^, ^49^, ^50^ Zhou et al reported that cancer‐directed surgery was a risk factor for committing suicide among patients with non‐small cell lung cancer, probably due to the increased frailty and lack of dignity in the postoperative period.^38^ Jayakrishnan and colleagues investigated the association between suicide and surgery. The patients with cancer who underwent high‐morbidity surgeries (30‐day overall postoperative morbidity > 30%) were more likely to commit suicide than those with low‐morbidity surgeries performed. In addition, no clear association of suicide with the anatomic site of cancer was obtained.^40^ Indeed, the patients usually suffered from general debility, and experienced depression, hopeless, and despair after surgery, which were risk factors of suicide.^40^ However, there was no consensus regarding the association between cancer‐directed surgery and suicide so far. Of note, suicide was a complicated phenomenon involving not only physiological but also psychological and social factors. Therefore, more factors should be taken into account to clarify this relationship.

Suicidal behavior in patients with cancer is affected by various factors. Compared with other causes of death such as accident, suicide is preventable.^1^ It is different to predict suicide, and therefore, more efforts should be made to improve the situation. Based on our findings, we suggest that kidney cancer patients with high risks of suicide should be considered for psychiatric evaluation. At present, several validated tools can be used for identifying depression risk, including the Beck Depression Inventory and the National Comprehensive Cancer Network guidelines.^13^, ^28^ Second, the patients at risk should receive psychotherapeutic interventions as soon as possible. It has been widely proved that psychotherapeutic interventions to the depression population can reap benefits in terms of reducing the suicide rate and improving the quality of life, such as participation in cancer support groups, stress management, and integrate psychological support into cancer care.^13^, ^27^ Additionally, the efforts for decreasing suicide need coordination and collaboration, including medical workers, family members even the whole society. Besides professional care, family communication and social support also play an important role in preventing suicides and suicide attempts.^1^, ^51^, ^52^

There were some limitations in our study. Besides pathological factors, suicide was affected by psychological and social factors which could not be provided by the SEER database. Moreover, there was also a lack of sufficient medical information in the SEER database, such as pharmacotherapy and genetic factors. Additionally, our study was based on the SEER database, which only collected the corresponding data of cancer patients in the US. Therefore, further study coving more countries should be conducted. Finally, data of failed suicide could not be obtained and exhibited, which might result in an under evaluation to suicide risk.

## 5 CONCLUSIONS

In summary, our study identified the independent risk factors of suicide for patients with kidney cancer. Diagnosis in early years (1973‐1992), male sex, unmarried status, non‐black race, higher histologic grade, and cancer‐directed surgery not performed were significantly associated with high risks of suicide. Whereas, age at diagnosis. SEER disease stage and radiotherapy did not relate to suicide. Thus, based on our study, clinicians could better screen and perform interventions to those with high risks of suicide, especially at vulnerable stages throughout diagnosis, treatment, and follow‐up. Further investigations are still needed.

## CONFLICT OF INTEREST

The authors declare that they have no conflict of interest.

## Data Availability

The data analysed during the current study are available from the corresponding author on reasonable request.
