# Supplementary material for: Risk factors associated with suicide among kidney cancer patients: A Surveillance, Epidemiology, and End Results analysis
Source: Cancer Med. 2019 Jul 11;8(11):5386–96. doi: 10.1002/cam4.2400 (PMC6718588; doi:10.1002/cam4.2400)
Supplement: Supplementary file 1 [file CAM4-8-5386-s001.docx]

Supplementary Table 1. Suicide rates and SMRs among patients with kidney cancer by demographic and clinic characteristics (1981-2015)

| **Variables** | **Suicidal death** | **Person-years** | **Suicide rate per 100,000 person-years** | ***P*** | **SMR**^†^ | **95% CI** |
| --- | --- | --- | --- | --- | --- | --- |
| **Total** | 198 | 917,106 | 21.59 | - | 1.81 | 1.58-2.08 |
| **Sex** |  |  |  |  |  |  |
| Male | 171 | 572,109 | 29.89 | **<0.001***** | 1.55 | 1.35-1.80 |
| Female | 27 | 344,997 | 7.83 |  | 1.60 | 1.05-2.32 |
| **Age at diagnosis** |  |  |  |  |  |  |
| ≤39 | 6 | 55,405 | 10.83 | 0.250 | 0.91 | 0.31-1.98 |
| 40-49 | 30 | 140,119 | 21.41 |  | 1.79 | 1.23-2.56 |
| 50-59 | 48 | 243,036 | 19.75 |  | 1.66 | 1.25-2.20 |
| 60-69 | 56 | 261,438 | 21.42 |  | 1.80 | 1.39-2.33 |
| 70-79 | 44 | 172,883 | 25.45 |  | 2.13 | 1.56-2.86 |
| ≥80 | 14 | 44,225 | 31.66 |  | 2.65 | 1.43-4.45 |
| **Marital status** |  |  |  |  |  |  |
| Married | 102 | 604,191 | 16.88 | **<0.001***** | 1.42 | 1.20-1.72 |
| Unmarried | 91 | 275,458 | 33.04 |  | 2.77 | 2.19-3.40 |
| Unknown | 5 | 37,457 | 13.35 |  | 1.12 | 0.36-2.61 |
| **Race** |  |  |  |  |  |  |
| White | 183 | 756,819 | 24.18 | **0.001**** | 1.83 | 1.59-2.11 |
| Black | 7 | 103,851 | 6.74 |  | 1.18 | 0.46-2.43 |
| Others | 8 | 52,144 | 15.34 |  | 2.21 | 0.94-4.36 |
| Unknown | 0 | 4,292 | 0 |  | - | - |
| **Histologic grade** |  |  |  |  |  |  |
| Grade Ⅰ | 16 | 124,084 | 12.89 | **<0.001***** | 1.08 | 0.61-1.76 |
| Grade Ⅱ | 67 | 354,020 | 18.93 |  | 1.59 | 1.23-2.01 |
| Grade Ⅲ | 45 | 149,397 | 30.12 |  | 2.52 | 1.84-3.38 |
| Grade Ⅳ | 12 | 24,766 | 48.45 |  | 4.06 | 2.09-7.10 |
| Unknown | 58 | 264,839 | 21.90 |  | 1.84 | 1.46-2.37 |
| **SEER disease stage** |  |  |  |  |  |  |
| Localized | 146 | 731,303 | 19.96 | **0.002**** | 1.67 | 1.42-1.97 |
| Regional | 30 | 140,827 | 21.30 |  | 1.79 | 1.26-2.55 |
| Distant | 18 | 33,178 | 54.25 |  | 4.55 | 2.73-7.19 |
| Unstaged | 4 | 11,798 | 33.90 |  | 2.84 | 0.74-7.28 |
| **Histologic subtype**^‡^ |  |  |  |  |  |  |
| cRCC | 82 | 418,517 | 19.59 | 0.341 | 1.64 | 1.28-2.04 |
| pRCC | 24 | 86,270 | 27.82 |  | 2.33 | 1.48-3.47 |
| chRCC | 10 | 37,833 | 26.43 |  | 2.22 | 1.06-4.07 |
| sRCC | 2 | 3,844 | 52.03 |  | 4.36 | 0.49-15.75 |
| cdRCC | 0 | 1,323 | 0 |  | - | - |
| Others | 80 | 369,319 | 21.66 |  | 1.82 | 1.51-2.26 |
| **Surgery performed** |  |  |  |  |  |  |
| Yes | 180 | 891,944 | 20.18 | **<0.001***** | 1.69 | 1.48-1.96 |
| No | 17 | 23,682 | 71.78 |  | 6.02 | 3.46-9.63 |
| Unknown | 1 | 1,480 | 67.57 |  | 5.66 | 0.07-31.51 |
| **Radiotherapy performed** |  |  |  |  |  |  |
| Yes | 4 | 10,526 | 38.00 | 0.296 | 3.19 | 0.70-8.16 |
| No | 194 | 906,580 | 21.40 |  | 1.79 | 1.57-2.06 |
| **Time from diagnosis** |  |  |  | 0.146 |  |  |
| 0-3 years | 93 | 375,986 | 24.73 |  | 2.07 | 1.64-2.54 |
| 4-6 years | 50 | 237,494 | 21.05 |  | 1.76 | 1.31-2.33 |
| 7-9 years | 33 | 144,090 | 22.90 |  | 1.92 | 1.36-2.70 |
| 10-12 years | 13 | 79,973 | 16.26 |  | 1.36 | 0.86-2.33 |
| >13 years | 9 | 79,563 | 11.31 |  | 0.95 | 0.50-1.80 |

^†^. Compared with the suicide rates of the general US population based on the Centers for Disease Control and Prevention’s Web-based Injury Statistics Query and Reporting System (1981-2015).

^‡^. cRCC = clear renal cell carcinoma, pRCC = papillary renal cell carcinoma, chRCC = chromophobe renal cell carcinoma, sRCC = sarcomatoid renal cell carcinoma, cdRCC = collecting duct renal cell carcinoma

* *P*<0.05, ** *P*<0.01, *** *P*<0.001
